# Effect of Accelerated Aging on the Sorption and Solubility Percentages of Silicone Facial Prostheses

**DOI:** 10.1055/s-0041-1731932

**Published:** 2021-10-21

**Authors:** Adhara Smith Nóbrega, Clóvis Lamartinede Moraes Melo Neto, Daniela Micheline dos Santos, André Pinheirode Magalhães Bertoz, André Luiz de MeloMoreno, Marcelo Coelho Goiato

**Affiliations:** 1 Department of Dental Materials and Prosthodontics, School of Dentistry, São Paulo State University, Araçatuba, São Paulo, Brazil; 2 Oral Oncology Center, School of Dentistry, São Paulo State University, Araçatuba, São Paulo, Brazil; 3 Department of Pediatric and Social Dentistry, School of Dentistry, São Paulo State University, Araçatuba, São Paulo, Brazil

**Keywords:** maxillofacial prosthesis, solubility, sorption, silicone elastomers, A-2186, MDX4-4210, polymers

## Abstract

**Objective** This study aimed to evaluate the effect of accelerated aging on the sorption and solubility percentages of the MDX4-4210 and A-2186 silicones.

**Materials and Methods** Two silicones (A-2186 and MDX4-4210) and three intrinsic pigments (bronze, black, and pink) were used in this study. Thus, six groups were created ( *n* = 10): Group 1 = bronze MDX4-4210; Group 2 = black MDX4-4210; Group 3 = pink MDX4-4210; Group 4 = bronze A-2186; Group 5 = black A-2186; and Group 6 = pink A-2186. The dimensions of all samples were the same (45-mm diameter (ø) × 1-mm thickness). The samples were aged for a total of 1,008 hours. In this period of 1,008 hours of accelerated aging, the sorption and solubility percentages of each sample were calculated at three time points (252, 504, and 1,008 hours).

**Statistical Analysis** Three-way analysis of variance (ANOVA) and the Tukey test were performed (α = 0.05).

**Result** Accelerated aging can significantly increase the sorption and solubility percentages of the MDX4-4210 and A-2186 silicones.

## Introduction

Congenital defects, trauma, or cancer can often cause facial tissue disfigurement, leading to distress, few job opportunities, bullying, and discrimination for people with this problem. 1 Therefore, facial defects can negatively affect the individual’s social, economic, and psychological well-being. 1

Facial prostheses may be used to rehabilitate patients with orofacial defects because they help to restore the patient’s self-esteem, quality of life, esthetics, and social life. 2 3 4 5 6 7 8 Silicone elastomer is the most used option to manufacture maxillofacial prostheses. 2 3 4 5 6 7 The advantage of this type of material is its ability to imitate human skin tissue both aesthetically and functionally. 1 4 The MDX4-4210 and A-2186 silicones are examples of two widely used silicones. 5 6 7 9

The degradation of the physical and mechanical properties of a silicone prosthesis is related to its exposure to ultraviolet rays, polluted air, and humidity, as well as its inadequate cleaning and daily handling. 2 5 6 9 The literature reports that the replacement of a silicone prosthesis occurs from 3 to 12 months after its manufacture due to its degradation. 2 3 6

Sorption and solubility of a material are clinically important characteristics. 2 The sorption represents the amount of water adsorbed on the surface of a material and absorbed by its body. 2 8 Therefore, in the sorption process, both adsorption and absorption occur simultaneously. 2 8 Solubility is represented by the solubilization of soluble compounds of a material and the loss of these components to the environment. 2 8

A silicone prosthesis may be in frequent contact with saliva, sweat, and/or water (due to hygiene of the prosthesis or rain). 2 8 Silicone is a material that can absorb water, saliva, blood and sweat, and it shows solubility. 2 8 10 It is important to mention that these factors (absorption and solubility) can affect the physical, mechanical, and chemical properties of a polymer (e.g., silicone elastomer). 2 8 10 Hulterström et al reported that if a prosthesis (e.g., silicone prosthesis) absorbs liquids to the point that it loses its original dimensions or shows solubility, the prosthesis may lose its functionality and appearance. 10

Based on the fact that there are no studies in the literature reporting whether accelerated aging can have an influence on the sorption and solubility of silicone elastomers, the objective of the present study is to evaluate the effect of accelerated aging on the sorption and solubility percentages of the MDX4-4210 and A-2186 silicones.

## Materials and Methods

### Formation of Groups

Six groups were created ( *n* = 10): Group 1 = bronze MDX4-4210; Group 2 = black MDX4-4210; Group 3 = pink MDX4-4210; Group 4 = bronze A-2186; Group 5 = black A-2186; and Group 6 = pink A-2186. The dimensions of all samples were the same (45-mm diameter (ø) × 1-mm thickness), 8 and they were manufactured by the same operator.

### Manufacturing of Samples

The A-2186 (Factor II, United States) and MDX4-4210 (Dow Corning Corporation Medical Products, United States) silicones were used in this study. In addition, the intrinsic pigments bronze (Tan FI - 215, Factor II, United States), black (Black FI - 205, Factor II, United States), and medium pink (Orbipasta, Orbital Colors, Brazil) were also used.

The silicones and intrinsic pigments were weighed on a digital analytical balance (Adventurer, Ohaus Corporation, United States). 2 3 4 5 6 7 8 The bronze pigment corresponded to 0.2% of the weight of each silicone. 2 3 4 5 6 7 8 The black pigment also corresponded to 0.2% of the weight of each silicone. 2 3 4 5 6 7 8 For the pink pigment, the pigments that constituted it corresponded to 0.122% (yellow), 0.006% (black), 0.03% (red), and 0.6% TiO _2_ (opacifier) of the weight of each silicone. 2 5 6 7 8

The industrial method of incorporating pigment into silicone was used. 6 7 8 In this method, the pigment and silicone were mixed by using a grinding machine (CHSG/3-Roll Mill, Chemieland, China). 6 7 8 Subsequently, the silicone was introduced into the metallic matrix, and it was closed and subjected to 1,000 Kgf for 10 minutes (Hydraulic press—Maxx, Essence Dental VH, Brazil). 3 7 8 The samples remained confined within the matrix under controlled temperature (27 ± 2°C) with their surface exposed to the environment for 72 hours to complete the polymerization of the material. 2 4 5 6 7 8 After this period, the samples were carefully separated from the matrix. 4 5 6 8

### Sorption and Solubility Assessments

To perform sorption and solubility assessments, the samples were subjected to a desiccation test initially and after each aging period according to the American Dental Association (specification 12). 8 11 For this procedure, the samples remained inside a desiccator (Odontobrás, Brazil) containing silica gel and at a temperature of 37 ± 2°C for 23 hours. 8 11 Subsequently, the samples were removed to a similar desiccator at room temperature (23°C) for 1 hour and then weighed on a precision digital scale (BEL Equipamentos Analítico, Brazil). 8 11 This cycle was repeated until the weight loss of each sample did not exceed more than 0.5 mg. 8 11 Therefore, the initial mass (W1) was reached when the difference between two successive readings did not exceed 0.5 mg. 8 Posteriorly, the samples were submitted to the accelerated aging procedure, and they were weighed one more time (W2). 8 Finally, the samples were submitted to a new desiccation and final weighing (W3). 8 The sorption and solubility percentages of each sample were calculated according to the following formulas 2 8 : Sorption (%) = (W2–W3)/W1 × 100; solubility (%) = (W1–W3) × 100.

The sorption and solubility assessments were performed by the same operator.

### Accelerated Aging

The accelerated aging of the samples was performed inside an aging chamber (Equilam, Brazil) according to the American Society for Testing and Materials, Designation G53–96. 12 The samples were subjected to alternating periods of UVB (ultraviolet B) light (UVB lamps 313, 40 Watts, Equilam, Brazil) and distilled water condensation saturated with oxygen under conditions of heat and 100% humidity. 2 3 4 5 6 7 8 Each aging cycle lasted 12 hours. 2 3 4 5 6 7 8 In the first 8 hours, the temperature was maintained at 60 ± 3°C, and the UVB light was imputed onto the samples. In the last 4 hours, the temperature was maintained at 45 ± 3°C and a condensation period occurred without UVB light. 2 3 4 5 6 7 8 This test was performed for a total of 1,008 hours. 2 3 4 5 6 7 8 In this period of 1,008 hours of accelerated aging, the sorption and solubility percentages of each sample were calculated at three time points (252, 504, and 1,008 hours). 5

### Statistical Analysis

All data were analyzed by using the Statistical Package for Social Sciences 20.0 (IBM Corp., United States). The Shapiro–Wilk statistical test was used to analyze the distribution of the numerical data. Then, three-way analysis of variance (ANOVA) and the Tukey test were performed (α = 0.05).

## Results

Tables 1 and 2 show the sorption and solubility percentages of each group at each time point (252, 504, and 1,008 hours). Tables 1 and 2 show that accelerated aging can significantly increase the sorption and solubility percentages of the MDX4-4210 and A-2186 silicones.

**Table 1:** Mean values (%) (standard deviation) of sorption of the evaluated groups

Silicones	Pigments	Time points		
		**252 h**	**504 h**	**1,008 h**
A-2186	Bronze	0.05 (0.03) Aa	0.07 (0.03) Aa	0.05 (0.02) Aa
	Black	0.03 (0.03) Aa	0.04 (0.03) Aa	0.04 (0.01) Aa
	Pink	0.03 (0.02) Aa	0.03 (0.04) Aab	0.07 (0.03) Ab
MDX4-4210	Bronze	0.10 (0.04) Aab	0.07 (0.03) Aa	0.12 (0.05) Ab
	Black	0.05 (0.04) Ba	0.05 (0.05) Aa	0.04 (0.03) Ba
	Pink	0.03 (0.03) Ba	0.14 (0.08) Bb	0.12 (0.07) Ab
Note: Tukey test; different uppercase letters in the vertical represent a statistically significant difference (for each silicone individually) ( *p* < 0.05). Different lowercase letters in the horizontal represent a statistically significant difference ( *p* < 0.05).				

**Table 2:** Mean values (%) (standard deviation) of solubility of the evaluated groups

Silicones	Pigments	Time points		
		**252 h**	**504 h**	**1,008 h**
A-2186	Bronze	2.00 (0.90) Aa	2.15 (0.87) Ab	2.23 (0.85) Ab
	Black	1.43 (0.34) Ba	1.51 (0.34) Ba	2.76 (4.01) Ab
	Pink	1.67 (0.66) ABa	1.77 (0.66) ABa	1.78 (0.66) Aa
MDX4-4210	Bronze	0.08 (0.61) Aa	0.46 (0.73) Ab	0.53 (0.75) Ab
	Black	0.43 (0.37) Aa	0.66 (0.39) Ab	0.74 (0.41) Ab
	Pink	0.43 (0.22) Aa	0.46 (0.23) Aa	0.60 (0.21) Aa
Note: Tukey test; different uppercase letters in the vertical represent a statistically significant difference (for each silicone individually) ( *p* < 0.05). Different lowercase letters in the horizontal represent a statistically significant difference ( *p* < 0.05).				

## Discussion

Based on Tables 1 and 2 , accelerated aging can significantly increase the sorption and solubility percentages of the MDX4-4210 and A-2186 silicones. Possibly, the degradation of these silicones caused by UVB light, oxygen, high humidity, and temperature variations generated these results. It is worth mentioning that UVB light can generate chemical changes in a polymer, 3 5 8 13 and this may have contributed to the increase in the sorption and solubility percentages of the MDX4-4210 and A-2186 silicones.

The photo-oxidative degradation of the silicones used in this study may be explained according to the following steps 13 14 15 :

1. Initiation step: Photodegradation of a silicone elastomer starts with the absorption of photons (UVB) by chromophoric groups. Polymers can contain intramolecular chromophoric groups and/or impurities containing chromophoric groups (intermolecular impurities). After the absorption of photons (UVB), the energy of the molecules increases and they assume an excited state, which leads to the breaking of bonds and the formation of free radicals.
2. Propagation step: Reaction of free polymer radicals with oxygen; production of polymer oxy- and peroxy- radicals and secondary polymer radicals, resulting in chain scission.
3. Termination step: Reaction of different free radicals with each other, resulting in crosslinking.

During accelerated aging, the samples were in a high humidity environment. Therefore, water may also have contributed to the degradation of the silicones used. According to Garcia-Fierro and Aleman, as water interacts with the chains of a polymer, it can produce some of the following effects in this order: (1) reorientation and chain displacement, that is, reversible loosening or effective plasticization of the structure; (2) solvation or reversible rupture of weak interchain bonds; and (3) irreversible disruption of the polymer matrix (microvoids). 16

A limitation of this study is that there are no criteria in the literature that report which percentages of sorption and solubility of silicone elastomers are clinically acceptable after a certain period of time. Further studies on the subject of the present study are recommended.

## Conclusion

Accelerated aging can significantly increase the sorption and solubility percentages of the MDX4-4210 and A-2186 silicones.
